# Service Allocators’ Experiences of Ethical Dilemmas and Strategies in Long-Term Care: A Qualitative Study

**DOI:** 10.1177/11786329241238883

**Published:** 2024-03-15

**Authors:** Ann Katrin Blø Pedersen, Marianne Sundlisæter Skinner, Maren Sogstad

**Affiliations:** Centre for Care Research, Department of Health Sciences in Gjøvik, Faculty of Medicine and Health Sciences, NTNU – Norwegian University of Science and Technology, Trondheim, Norway

**Keywords:** Needs assessment, long-term care, resource allocation, elderly care, ethical dilemmas, qualitative research

## Abstract

The provision of long-term care services for older adults is characterised by increasing needs and scarce resources, leading to ethical dilemmas. This qualitative study explored the ethical dilemmas experienced by healthcare professionals when allocating long-term care services to older adults and the strategies used to handle ethical dilemmas. Data from semi-structured individual interviews, focus group interviews, and observations of service allocators assessing needs and assigning long-term care services to older adults were analysed using content analysis. The overarching theme was the struggle for safe and equitable service allocation. The identified dilemmas were: (i) Struggles with A Just Allocation of Services due to Limited Time and Trust, (ii) Pressure on Professional Values Concerning Safety and Dignity, and (iii) Difficulties in Prioritising One Group Over Another. The strategies to deal with ethical dilemmas were: (i) Assessing Needs Across the Entire Municipality, (ii) Ensuring Distance to Service Recipients, (iii) Working as a Team, and (iv) Interprofessional Decision-Making. Scarce resources, organisational limitations, and political expectations drive the ethical dilemmas in long-term care service allocation. An open public discussion regarding the acceptable minimum standard of long-term care is needed to reduce the ethical pressure on service allocators.

## Background

Long-term care for older adults is on the political agenda in all industrialised countries because of the challenges related to demographic changes.^1,2^ Consistent with political aims, efforts have been made to reduce the use of specialist healthcare, leading to shorter lengths of hospital stays.^3-6^ Consequently, long-term care services have been given responsibility for more patients with severe conditions and needs.^7^ These changes increase the demand for long-term care services and enhance the need for priority setting in the sector. Upon application for services, municipal service allocators are responsible for assessing care recipients’ needs and making individual decisions on the amount and content of long-term care services provided.^8^ Ethical issues and dilemmas are an inherent and inseparable part of being decision-makers in long-term care services.^9,10^ An *ethical dilemma* is defined *‘*as a situation in which a choice has to be made between at least 2 options, none of which resolves the situation in an ethically acceptable way’^11^ (p. 661). Dilemmas occur when reality is inconsistent with professionals’ intentions to provide safe and equitable care,^12^ so they must compromise or act against professional values,^13^ often causing moral distress^14^ or cognitive dissonance.^15^ Cognitive dissonance concerns the personal tension experienced when individuals’ actions contradict or are inconsistent with their values or beliefs.^15^ These compromises are often a result of institutional constraints,^14^ personnel shortages,^16^ and limited time to provide necessary care due to limited resources.^17,18^

Healthcare professionals have reported justifying priorities in eldercare that are inconsistent with their professional values due to organisational demands.^19^ Incongruence between service allocators’ desire to conduct comprehensive and needs-led assessments of care recipients with dementia and their practice has been reported, where psychological needs were omitted from the assessments.^20^ A method of supporting healthcare professionals who face ethical dilemmas in various long-term care settings includes implementing ethics reflection groups that enhance awareness of ethical aspects, professional development, and an understanding of and respect for patients.^21^ Such initiatives have been shown to improve handling of ethical dilemmas, increase employee cooperation, and increase service quality. Support from stakeholders, available time, knowledge and skills in ethics, and access to supervision are essential factors to sustain this in practice.^21^ However, organisational demands for cost-efficiency and standardisation, including increasing task- and rule-oriented work and time constraints, interfere with the quality of care.^22^ Long-term care service allocators participating in ethical work groups struggle to balance their moral responsibility to act in the best interest of the care recipients and the expected accountability concerning control and efficiency in their professional role.^18^

As in several Western countries, the Norwegian health and social care system is based on a liberal welfare model funded from the state budget.^23^ The overall intention of the public organisation of health and care is to establish a just allocation with equal access to services and transparency in decision-making.^5^ The gradual reduction of specialist care is driven by a decentralising care and treatment policy,^7^ based on ideas from New Public Management (NPM) where service and effectiveness are central.^24^ Following ideas from NPM, many Norwegian municipalities provide long-term care services in a purchaser-provider model, splitting the allocation and provision of long-term care services into 2 separate units.^8,22,25^ This model draws on standardisation and formality to guide service allocation and builds on the idea that formal contracts guide the relationship between long-term care providers and care receivers.^22^ In the first step, service allocators within an allocation unit assess the appropriate long-term care based on care needs related to specific criteria and gather and provide information before specifying contracts and ordering services. In the second step, the long-term care provider unit is responsible for delivering services specified by the service allocators.^22^ The long-term care service allocators are central to implementing policies and have a profound influence on older adults’ access to care.^26^ Marketisation of the health and care system has prioritised economic concerns and cost-effectiveness over ethics.^27^ The insufficient transfer of resources to the long-term care service may hinder the allocation of safe care to older home-dwelling care recipients,^7,10,28^ increasing service allocators’ experience of ethical dilemmas.^18^

With increasing pressure on long-term care resources, ethical dilemmas will multiply in the future. Therefore, increasing the understanding of these dilemmas and how they are addressed in practice is essential. This study aimed to explore the ethical dilemmas experienced by health professionals allocating long-term care services to older adults, and the strategies used to handle ethical dilemmas.

## Methods

In this qualitative study, using Norway as a case study, we combined data from individual and focus group interviews and observations.

### Data

Invitations to participate in the study were sent to the heads of healthcare services in 3 selected municipalities to recruit service allocators. Thirteen service allocators were informed about the study and provided consent to participate. All participants were women. Nine participants were nurses, 2 physiotherapists, and 2 occupational therapists. Two municipalities had a purchaser-provider model, while the third had a split model where the informants worked half the time as service allocators and the other half as service providers.

Nine semi-structured individual face-to-face interviews were conducted. The individual interviews lasted between 45 minutes and 1 hour. The interview guide for the individual interviews was structured with questions about service allocators’ experiences regarding considerations, dilemmas, and collaboration in the needs assessment process and the allocation of services to older adults.

The study involved the observation of weekly allocation meetings where the allocation of services for individual users was discussed, mainly regarding which older adults should receive nursing home stay. The participants in these meetings were either service allocators or service allocators *and* long-term care staff, such as nurses, physiotherapists, occupational therapists, and leaders of nursing homes. The first author conducted observations at 14 meetings across the 3 municipalities. The meetings lasted for 60 to 90 minutes. The observations focussed on what the meeting participants emphasised when discussing the allocation of long-term care services to older adults. The participants were asked questions before and after the meetings to clarify issues arising during the observations. Additionally, 140 hours of observations of service allocators’ daily practices were used. Field notes were taken, and any data that could identify patients were excluded from the notes.

In addition, one focus group interview was conducted in the medium-sized and one in the large municipality, with 5 and 6 participants, respectively. All the participants from the individual interviews took part in the focus group interviews, and they were joined by 1 and 3 additional participants from their allocation teams. In the small municipality, there were only 2 service allocators, so no focus group interview was conducted. The 2 focus group interviews lasted between 60 and 90 minutes. Both the individual interviews and the focus group interviews were conducted by the first author at the service allocators’ workplaces. The focus group interviews were conducted after the individual interviews and observations were concluded in each municipality. Their aim was to clarify the dilemmas that emerged as salient in the individual interviews and observations. While the individual interviews served as the primary data source in the analysis, supplementary information was added from field notes and focus group discussions to deepen the researchers’ understanding of the context and the service allocators’ evaluations, decisions, and dilemmas.

### Disclosures and ethics

The study was guided by the ethical standards of the 1964 Declaration of Helsinki and its subsequent amendments. Ethical approval was granted by the Regional Committee for Medical and Health Research Ethics, REK North (2020/111946), and the Norwegian Centre for Research Data (NSD; reference number 693007). Written informed consent was obtained from all participants before their participation in the study, and service allocators were informed that they could withdraw from the study at any time. Confidentiality has been maintained throughout the study, including reporting findings.

### Analysis strategy

All interviews were audio recorded and transcribed verbatim. All interviews and field notes were closely read, and these texts were systematically condensed into meaningful units and codes related to the ethical dilemmas experienced by service allocators when assessing and allocating services to older adults. NVivo 20 software was used to organise, review, and analyse the meaning units and codes. We then determined categories and identified themes, using qualitative content analysis.^29^ First, we examined the interviews, starting with the individual and then the focus group interviews, taking into account the entirety of the context when condensing and labelling meaning units with categories closely aligned to the text. This process was followed by identifying the latent content of the codes through the formulation of sub-themes. We applied a similar approach to the field notes. Initially, we segmented the field notes into meaning units and, accounting for contextual nuances, synthesised them into categories closely aligned with the original text. However, given the inherent presence of researchers’ observations and interpretations within the field notes, certain sections exhibited concentrated informational content. Therefore, only where feasible were these observations abstracted into interpretations aimed at elucidating the latent content, uncovering underlying meanings. The condensed meaning units from all the texts, interviews and observations were viewed as a whole before they were abstracted into sub-themes. The interviews and observation notes were reread several times to ensure that there were no contradictions in and between sub-themes. The first author conducted the first steps of the analysis, whereas all 3 researchers were involved in determining the main theme and sub-themes. In the final step, all authors critically reviewed the themes and re-read codes and categories, ensuring a good balance between closeness and analytical distance to the data^30^ and a joint understanding and interpretation of the results.

## Results

This study explored the ethical dilemmas experienced by service allocators and the strategies used to handle them. The overarching theme was ‘The Struggle for Safe and Equitable Service Allocation’. The inherent conflict between the needs of older adults and the scarcity of long-term care resources in municipalities was critical. The ethical dilemmas we identified were: (i) Struggles with A Just Allocation of Services Due to Limited time and Trust, (ii) Pressure on Professional Values Concerning Safety and Dignity, and (iii) Difficulties in Prioritising One Group Over Another. The strategies to deal with ethical dilemmas included: (i) Assessing Needs Across the Entire Municipality, (ii) Ensuring Distance to Service Recipients, (iii) Working as a Team, and (iv) Interprofessional Decision-Making (Table 1).

**Table 1.** Analysis scheme showing the ethical dilemmas experienced by service allocators.

Main theme	Theme	Sub-theme	Meaning unit	Text
The struggle for safe and equitable service allocation.	Ethical dilemmas in service allocation.	Struggles with a just allocation of services due to limited time and trust.	The service allocators experienced dilemmas in the fair distribution of services due to limited time to uncover older adults’ needs and limited trust in service providers’ needs evaluations.	And for completely new patients, we try to go out for assessment visits to the best of our ability. But often the service must start prior to our visits. So, often, it is home nursing that perhaps, for example, travels there first and maps the need. [. . .] We have so little capacity now, but the goal, our wish, was that we could do more assessment visits ourselves. [. . .] to look at alternative things. People don’t really know what they apply for, either. And often, there are several solutions. (M2, I5)
				Then, it may happen that the most important information is almost missing. But we’re detectives like that, so we use the patient record and [. . .] search thoroughly. So, we can get a lot of good information from the hospital. But sometimes it is awful [the information], and we don’t manage to find out anything more than just . . . that, it’s so limited that you barely get to write what you need to, then we just have to sort it out. But we do tell the service providers to update [the information]. But then there are times when you don’t have time to wait. (M1, I2)
		Pressure on professional values concerning safety and dignity.	The ethical standard for safe and dignified services as opposed to legally justifiable minimum standard services.Experienced dilemmas of being unable to allocate more services regarding the older adults’ unmet needs, caused an allocation practice inconsistent with ethical and professional values.	[. . .] there is a reason why we grant long-term placements. Because it is considered that necessary and safe health care cannot be provided at home. So, then it is very difficult to defend that it takes so long before they get the place they need. So that is terribly difficult. And then we must try to put in more help in the meantime before allocating that place. But it’s not always possible to help everyone either [. . .] It’s not ok that it’s like that. But there is little we can do about it when there are no beds available for them. (M3, I9)
	
				We must take safety into account in each case, but we are pushing it quite far. (M3, GI, I8) It feels like we are on the edge sometimes. . . (M3, GI, I10) Yes. Try things out until the bitter end almost. . . (M3, GI, I8) It is often not okay. (M3, GI, I9) We try things that we know won’t work, but we try one more time anyway. (M3, GI, I11)
		Difficulties in prioritising one group over another.	Experienced tension between political expectations and the ethically justifiable prioritisations of service allocation to groups of older adults.	I think it has always been like that, but the cut in nursing home places should [enable us to put] more focus on prevention and coping. It is, after all, in line with the prevention and coping strategy [political] to cut nursing home places and strengthen home services. [. . .] I hope we have more resources for prevention in a few years than we have right now. (M1, GI, I1)
				And then there is this issue of seeing the whole picture. Because I think that within mental health and substance abuse care, at least in mental health, they do a lot of prevention. They start with low-threshold services. But that means that they invest less in the people who are in a worse state. And at least the way it seems to go, much more is left to the home nursing services, who have to step up and take care of them. I do think there is a lot of psychiatry within the home nursing services now, who don’t get follow-up by psychiatric healthcare because they have their focus on early prevention. And then the [patients] just get worse, because they [the home nursing services] don’t have the competence, [and] they don’t really have time for them either, to prioritise. So then there are some groups [of patients] that don’t fit in and that maybe get worse because they invest in early effort in a few [patient groups]. (M3, GI, I10)
				Now, we have a room that we use for preventative short-term stays. Because we think that hospital admission might be avoided and that we prevent [care recipients] from getting worse. So, we have tried to have one or two beds at the nursing home, which we have used for prevention, because some people may benefit from a short stay, a planned stay for preventative purposes. [. . .] But then there is this issue with pressure from the hospital and the payment from there, [. . .] and it’s hard to think preventively, while at the same time dealing with the pressure from [the hospital and] those who already are in a very bad condition. (M3, GI, I10)
	Strategies to deal with ethical dilemmas.	Assessing needs across the entire municipality.	More equitable distribution of services with a separate and neutral allocation unit with an overall perspective of needs across the entire municipality.	I think that the injustice that you can minimise by creating a unit like ours, that serves the entire municipality, is precisely that geographical injustice [. . .], and we have stopped it as best we can by having this unit. Where we are not sitting on a bag of money and a budget [and we don’t have to deal with] the complications of temporary contracts, and we’re not close to the user either. (M1, GI, I4)
		Ensuring distance to service recipients.	Fairer needs assessment with distance to the care recipients, contributing to objectivity.	We don’t really have a personal relationship to those who should get and shouldn’t get services. Those who should have more and those who should have less. So, we have no hidden agenda when we allocate services, and [the distance to the care recipients] generates at least a greater safeguarding of the principle of justice. (M1, GI, I4)
		Working as a team.	Critical discussions of the allocation criteria contribute to more equitable allocation practices.	What I view as a strength when we discuss cases is that it is not the [family member] who shouts the loudest who gets their [next of kin] into the nursing home, even if you are their case manager, right? We have this unit and criteria to follow. So, it’s not just from my head what kind of services the person gets. It is discussed here. (M3, GI, I7)
		Interprofessional decision-making.	Using other professionals with different expertise to compensate for lacking competence and contributing to a more equitable allocation.	So, professional development, you get that through courses and perhaps meetings with other professionals, such as dementia coordinators, doctors, physiotherapists, and other professionals. And we also have, when it comes to case management, we have also had a lawyer here. We need that, absolutely. Perhaps we need that the most, more than <laughs> dementia issues. Yes, I have much to learn [about the legal side of things] about case management, rights, and safety, and what [citizens] are actually entitled to. That’s quite a lot. (M3, I8)

Abbreviations: M, municipality; GI, group interview; I, informant.

### Ethical dilemmas in service allocation

#### Struggles with a just allocation of services due to limited time and trust

The service allocators emphasised that a just assessment should be neutral and based on older adults’ needs, including both individual service needs and the needs of older adults in the entire municipality. However, they experienced issues with service allocation due to limited time to identify older adults’ actual needs and limited trust in service providers’ evaluations of older adults’ needs. Service allocators needed to receive comprehensive information from service providers about older adults’ situations to allocate the right service to the right care recipient at the right time. Allocations were based on information about older adult cases from multiple sources, such as home-nursing staff, home-care staff, general practitioners, relatives, and care recipient’s self-reports. Limited time resulted in the service allocators rarely being able to meet potential new care recipients more than once before service allocation. After this meeting, service allocators cooperated with service providers to gather information about older adults’ service needs. Collecting information was time-consuming and, due to limited time, could result in misconceptions and situations where the care recipient needed more or less assistance and follow-up than what was allocated. Time constraints also caused service allocators to be reliant upon service providers’ evaluations, potentially causing concerns regarding how justly the service allocations aligned with the needs of older adults.

Service allocators described different perspectives between themselves and service providers when assessing older adults’ service needs. Service providers were primarily concerned with the individual needs of older adults. In contrast, service allocators had a broader perspective, considering the totality of needs within the municipality before allocating services. The service allocators were concerned that evaluations of older adults’ needs had great variations due to service providers’ perceptions and that some service providers advocated for older adults, threatening equitable service allocation. One service allocator elaborated:

*And then we trust* [the service providers’] *decisions. And that’s a good question: Should you trust a nurse? It’s like, that nurse, does she view the glass as half full or half empty?* [The perspective depends on the] *individual, and you put a lot of trust in the nurse’s written account. Do* [they] *only describe what doesn’t work, or do they also describe what does work?* (M1, I1)

### Pressure on professional values concerning safety and dignity

Limited long-term care resources caused an allocation practice that contradicted ethical and professional values and was seen as insufficient to maintain the safety and dignity of older adults. Overall, older adults’ service needs exceeded the available resources, resulting in unmet needs and creating an ethical dilemma between providing the legal minimum necessary service and professional ideals that require more than the minimum level of service. Although the allocation practice was generally considered legally justifiable, service allocators were occasionally concerned with older adults’ safety, dignity, coping, and quality of life. They expressed concerns, particularly regarding home-dwelling frail older adults who were either waiting for a nursing home vacancy or were discharged after a short-term nursing home or hospital stay. The lack of available nursing home places led service allocators to increase the provision of home care services to the limits of what was manageable for service providers and safe and dignified for care recipients. Service allocators expressed concerns regarding their own safety evaluations of home-dwelling older adults, noting that situations that had shocked them at the beginning of their careers as service allocators no longer had the same effect. Despite that, several of the service allocators explained that they felt concerned about care recipients’ safety and struggled with decisions they had to make. One service allocator cried when she described a home-dwelling older adult who was on a waiting list for a nursing home place and needed assistance with toileting:

*So, he presses the alarm when he knows he needs to go to the bathroom. Then it can often take half an hour before home nursing arrives* [. . .] *And then the man gets desperate, tries to go to the bathroom, and he doesn’t get there. And then he falls, and lying on the floor, he does it in his pants and feels so ashamed. And then the home care* [worker] *comes and says it’s no problem.* [The home care worker] *knows this very well.* [. . .] *But he. . . doesn’t want it to be this way. And I think that’s awful. It’s really awful.* (M3, I9)

Limited long-term care resources also cause dilemmas because older adults’ expectations of service allocation exceed the available resources; thus, allocation practices were inconsistent with the wishes of older adults, raising service allocators’ concerns about older adults’ dignity. For example, service allocators sometimes had to demand that frail older adults found solutions to address their own care needs or made decisions that were against their own wishes, impacting negatively on their quality of life. One dilemma was whether service allocators should demand older applicants of care services, who for example had been widowed, to take care of tasks in the home that they had never done before and did not want to do. Another dilemma involved requiring older adults who lived in 2-floor homes but who were unable to climb the stairs to their bedroom, to move the bed to the living room, even if the older adults perceived it as undignified or if they did not want such an arrangement. One service allocator elaborated:

*And if you then have a house on two levels, and you don’t have a bedroom on the ground floor, but* [you] *have a bathroom there, for example. Then you must live for a long time having a bed in the living room. Can we sort of demand people to do that? Because, in a way, it is not the municipality’s fault that people live like that.* [. . .] *And then dignity comes into play with this, there are a lot of people that will object to this.* (M3, I7)

### Difficulties in prioritising one group over another

Political policies provide guidelines regarding priority setting that service allocators may disagree with, causing ethical dilemmas. Cuts in nursing home capacity were justified by a policy shift towards coping, prevention, and early intervention. However, these cuts increase the pressure on home care services, so service allocators experienced ethical dilemmas because the municipalities did not receive more resources in home care services, consistent with these political expectations. One example given by service allocators was that the high number of home-dwelling older adults with severe functional decline in the municipality, that is, home care recipients in need of a nursing home place, made it hard to justify granting older adults with a higher functional level preventive nursing home stays. Thus, service allocators were torn between political expectations and the safety of frail older adults when allocating services.

In one of the focus group interviews, service allocators discussed the correct use of different types of services in the municipality and the contradictions between their experiences and local policies. For example, they discussed how they had a practice of allocating sheltered housing to older adults with dementia, which was in line with the goal of local policies. However, this practice was at the expense of care recipients with mental health concerns or substance abuse. The service allocators argued that older adults with dementia should be in nursing homes due to being especially vulnerable to several moves, leaving sheltered housing available for persons with mental health concerns or substance abuse. Nevertheless, the service allocators explained that the latter group of older adults were often not prioritised for services, which they considered to be unfair. Additionally, the home care service lacked the skills and/or time to provide care for the latter group, leaving them at risk of unmet needs. The dilemma is illustrated in the following quote from the focus group interview:

*Because we don’t have enough sheltered housing, we must sort of look at what kind of groups we see living in sheltered houses in the future. We have tried this quite a bit within dementia care. So, that is a discussion to take, is it right or not.* [. . .] *I think that when it comes to mental health, it’s a bit more difficult for me because it’s not that concrete, and we’re talking about people aged 50 and over. That they have sort of been given up.* [. . .] *Because the specialised health services don’t think they belong there, and we don’t know where they belong in the municipality either. So, they are a group that. . .* (M3, GI, I10).

*Who is treated unfairly, yes. . .* (M3, GI, I8)

### Strategies to deal with ethical dilemmas

We identified several different strategies that allocators used to deal with ethical dilemmas in needs assessment and allocation of services.

### Assessing needs across the entire municipality

In the 2 municipalities that had implemented the purchaser-provider model of service allocation, this was viewed as a way to ensure a fairer distribution of services, as it ensured distance between the decision-making and operative branch. The service allocators viewed themselves as more neutral and in tune with the needs of older adults across the entire municipality compared with service providers, who were viewed as more subjective and individual-oriented. A general view among all the informants, including those from the third municipality, was that allocation units enable setting priorities based on needs rather than circumstances or strong-willed relatives. The service allocators from the 2 municipalities that had implemented the purchaser-provider model indicated that allocations were more equitable compared to before the transition to this organisational model. Before the transition, there had been greater variation in service allocation between municipal districts.

### Ensuring distance to service recipients

Service allocators from all 3 municipalities expressed that having an allocation unit enabled more neutral evaluation of needs because allocators could maintain their distance to care recipients. In the municipalities with a purchaser-provider model, service allocators avoided forming personal relationships with care recipients, which contributed to increased objectivity and fairness (equity) in the assessments and allocations. In the third municipality, service allocators talked about being affected by their personal relationships with care recipients when making allocation decisions.

### Working as a team

The burden of decision-making in difficult ethical dilemmas was reduced through the support of other service allocators through teamwork. Furthermore, to enhance equity and reduce subjective assessments, service allocators emphasised how they relied on being able to discuss allocations in these teams. Team discussions were seen as important, as parallels could be drawn to previous similar cases to increase equitable and appropriate allocations. Each municipality followed specific criteria for service allocation to older adults. However, participants reported that understanding and interpreting several criteria for service allocation was challenging. Thus, they emphasised the importance of being a team for handling ethical dilemmas and enhancing critical questions regarding the allocation criteria. One service allocator explained:

*Because I think if you hadn’t had those discussions and we each had to assess whether someone fulfils the criteria for a long-term placement or sheltered housing or a home adapted for round-the-clock care. I think that would have been terribly difficult because those criteria are not easy to navigate by. So, there we are somewhat dependent on good professional discussions.* (M3, I9)

### Interprofessional decision-making

Service allocators collaborated with other municipal professionals with different expertise in, for example, dementia, cancer, and the legal system to handle ethical dilemmas regarding older adults’ needs and ensure that their legal rights were met. This was especially important in cases in which service allocators felt that their competence was insufficient to make appropriate allocation decisions. These inter-professional collaborations contributed to improving the knowledge related to older adults’ needs and the accuracy of decision-making in the allocation of services.

## Discussion

This study provided insights into the ethical dilemmas that service allocators experience when reasoning and justifying their decisions regarding the allocation of long-term care services to older adults. Our overall findings suggest that older adults’ needs for care exceed long-term care resources, causing service allocators to be stuck between different ethical values considering the fairness, safety, and dignity in older adults’ situations. The study’s findings also show that service allocators use different strategies to handle these ethical dilemmas.

The service allocators in this study felt a strong obligation and responsibility to justly allocate long-term care services to older adults. This is in accordance with the purchaser-provider model, where a primary function of the service allocator role is fair distribution of the welfare state’s resources.^31^ Our findings show that service allocators experience dilemmas between providing the legal minimum service and allocating services fairly and sufficiently to maintain older adults’ safety and dignity. Over the last few decades, several Norwegian healthcare services have been regulated by law. The Patient and User Rights Act^32^ and Health and Care Services Act^33^ are examples of the legal basis for assessing individuals’ rights to long-term care services. These laws add frameworks for service allocation that are occasionally quite detailed.^34^ However, fairness and equity in service allocations are challenging due to uneven development of individual rights and choices, with patterns of inequity where not all patient groups are equally heard or responded to.^9^ Results of this study show that groups of older adults with mental health concerns or substance abuse are often not prioritised for healthcare services, whereas older adults with dementia are. This is in line with findings from other studies that show that after the introduction of NPM, healthcare professionals experienced less time to address mental health needs^35^ and that older adults with mental health problems are a particularly vulnerable group.^36^

Further consequences of the adoption of NPM strategies in long-term care services are less flexible services, rationing of care, increased reporting and documentation requirements, detailed goal management, and reduced time for conversations, basic needs, caring, and the nurse-patient relationships.^16,35-38^ However, some of our findings indicate that the fairness of service allocation to older adults is enhanced by having allocation units, making the assessment and allocation more neutral and equal across the entire municipality. The strategies involving working in teams and engaging in interprofessional decision-making in needs assessments can reduce ethical dilemmas and enhance knowledge regarding older adults’ needs and legal rights to long-term care services. However, our findings also show that service allocators can struggle to uncover the needs of older adults. This struggle is partially due to organisational boundaries, such as the lack of resources and formalised, task-oriented practice, which also challenges patient involvement in the allocation process.^39^ Having a separate allocation unit causes limited contact with care recipients and limited time to collaborate with service providers, which, according to our findings, can cause incorrect and unjust allocations.

The standardisation and formalisation of long-term care allocation with the purchaser-provider model,^22^ is criticised for minimising opportunities for professional and ethical judgement in need assessments.^40^ Consequently, service allocators, as shown in this study, struggle to balance ethically acceptable service allocation to meet individual needs with organisational demands for cost-efficiency.^41^ Organisational structures are often the source of ethical dilemmas experienced at work, particularly when healthcare personnel, due to structural constraints, cannot act according to their professional convictions and values.^11^ Our study shows that service allocators make decisions that are at odds with their ethical and professional values. When assessing services for older adults who are only marginally functioning in their homes, service allocators feel caught between the political goal that older adults should remain in their homes for as long as possible and the older adults’ safety and dignity. Insufficient nursing home capacity leads service allocators to grant more home care services to safeguard older adults in their home. However, often these ‘top-ups’ of home care, granted as a care recipient is waiting for a nursing home placement, conflict with the allocators’ own professional judgement of what is considered safe and dignified. Additionally, service allocators experience ethical dilemmas regarding prioritisation of preventive services. The deficit in nursing home capacity makes it hard for service allocators to justify using available places in nursing home for preventive stays because of the many older adults with severe functional decline. Thus, the political expectations of cost-efficiency strategies contradict what service allocators perceive as ethically justifiable allocations of long-term care services to older adults.

When service allocators’ actions are inconsistent with their values and/or beliefs, they experience cognitive dissonance in trying to meet 2 conflicting demands simultaneously.^15^ This dissonance causes discomfort for the individuals experiencing it, leading them to rationalise their actions to reduce the discomfort. Festinger^15^ propose that individuals can reduce dissonance through multiple pathways. Two alternatives include the individuals changing themselves by altering their attitudes and beliefs about their actions, or the individuals changing their circumstances—for example, by removing themselves from the situation. If healthcare professionals are systematically prevented from meeting care recipients’ need for care, due to the above-mentioned structural constraints, they will potentially face burnout and desire to leave the profession.^13^ Such problems can become especially noteworthy for those providing services that have close relationships with care recipients. Owing to the worldwide shortage of nursing workforce,^42^ the moral concerns raised in this study must be taken seriously.

Our findings show that older adults’ needs are not always adequately met in service allocations, causing several ethical dilemmas concerning older adults’ safety and dignity. The International Council of Nurses (ICN) states that respect for human rights, including cultural rights, dignity, the right to choice, and the right to be treated with respect, is inherent to nursing.^43^ Furthermore, human rights involve the right to a universal minimum standard of healthcare.^44^ However, a concrete description of this right to healthcare is not provided, generating debates related to the interpretation and application of this right.^45^ Clarity regarding the minimum standard of long-term care is both an ethical and legal concern.^45^ Our findings show that despite the service allocators’ strategies for handling ethical dilemmas and individuals’ rights to long-term care services being regulated by law, they experience the allocation of services as morally unacceptable regarding older adults’ safety, which is ethically and legally concerning. Service allocators would benefit greatly from a more open public discussion about what needs the public services *can* and *cannot* meet in the current and future long-term care climate. There is a need for an open discussion about what constitutes an acceptable minimum standard of long-term care for older adults, one that complies with care recipients’ legal rights and healthcare professionals’ ethical values and norms. Currently, there is no mutual understanding between healthcare practitioners, the general public and politicians regarding the scope of services the welfare state can offer. A willingness of politicians to openly declare the limitations of public services provision can alleviate the burden on service allocators, allowing them to operate more effectively and reducing the (ethical) pressure they currently face. However, any set of minimum standards must be adjusted according to the needs of older adults in different settings, contexts, and countries.^45^

### Methodological considerations

In this study, a qualitative approach was used to elucidate the ethical dilemmas experienced by health professionals who allocate long-term care services to older adults, and the strategies used to handle ethical dilemmas. A strength of this study is the use of data from several sources, including individual interviews, focus group interviews, and observations, which is a confirmation strategy to increase study validity. The individual interviews were the main source of data and were conducted to give the participants time and comfort^46^ to express their experiences with ethical dilemmas and strategies in service allocation. The focus group interviews and observations were used to support and validate the findings from the individual interviews. However, focus group interviews also contributed to acquiring different kinds of insights, for example into the collaboration between several people.^47^ The focus group interviews also provided insight into topics not covered by the individual interviews, such as dilemmas regarding older adults with mental health issues. Challenges in analysing data from several sources was handled by involving all 3 authors in several discussions regarding the abstraction of main units into sub-themes. Having included all 3 authors in the analysis of the data strengthened the dependability and consistency of the findings^48^; as did the use of interview guides in the data collection phase.^49^

The informants mostly represented the nursing profession and were women. Since the allocation service is primarily staffed by women, and nursing is a common profession,^50^ we considered the sample representative of the study setting. However, this study’s relevance to other contexts may be restricted by variations in the service settings of long-term care in different countries. Nonetheless, since healthcare professionals’ experiences with ethical dilemmas in priority setting is a challenge that is not specific to Norway but is a phenomenon that occurs in health systems across the world, we believe that the study and its results are relevant to other countries.

## Conclusions

Service allocators feel a strong obligation and responsibility to provide safe and equitable allocation of long-term care services to older adults. However, scarce resources, organisational limitations, and political expectations for cost efficiency contradict service allocators’ evaluations of the safe and equitable allocation of these long-term care services. These divergent expectations cause ethical dilemmas and service allocations that go against ethical and professional values, causing older adults’ needs for safety and dignity to be inadequately met. An open public discussion regarding what should be the acceptable minimum standard for long-term care, compliant with citizens’ legal rights and healthcare professionals’ ethical values and norms, is needed to help ensure older adults’ safety and dignity and guide service allocators in future service allocation.
